# Tradition Mills' Picholine Olive Oil Physicochemical Characterization and Chemical Profiling across Different Cities in Morocco

**DOI:** 10.1155/2020/1804723

**Published:** 2020-09-17

**Authors:** Hamza El Moudden, Yousra El Idrissi, Chakir El Guezzane, Walid Belmaghraoui, Adil El Yadini, Hicham Harhar, Mohamed Tabyaoui

**Affiliations:** ^1^Laboratory of Materials, Nanotechnology and Environment, Faculty of Sciences, Mohammed V University of Rabat, Av. Ibn Battouta, B.P. 1014, Rabat, Morocco; ^2^Laboratory of Spectroscopy, Molecular Modeling, Materials, Nanomaterials, Water and Environment, CERNE2D, Faculty of Sciences, Mohammed V University of Rabat, Av. Ibn Battouta, B.P. 1014, Rabat, Morocco

## Abstract

This study aims to determine the quality of olive oils (Picholine variety) from the traditional oil mills in different Moroccan cities by means of physicochemical characterization and chemical compositions. All samples of olive oil were collected from traditional oil mills. Physicochemical analyses of free fatty acid (FFA), iodine value (IV), saponification value (SV), specific extinction at 232 and 270 (E232, E270), chlorophyll content, carotenoid content, fatty acids (FAs), and total phytosterols composition were performed with respect to the International Olive Council (IOC) standards. These oils were revealed to be rich in unsaturated fatty acids (UFAs): C18 : 1, C18 : 2, and C18 : 3, and that the total phytosterols content ranged between 142.68 and 208.72 mg per 100 g of oil. Also, the chlorophyll contents, for most of the studied samples, are less than 2 mg/kg, while the carotenoid content varied between 0.13 and 0.63 mg/kg. These results, along with the physicochemical assays, helped classify the oils studied into three categories: extra virgin, virgin, and ordinary virgin olive oils. These results confirm that the conditions under which olive oils are collected, pressed, and stored influence the quality of the oil produced. Therefore, there is a need to inform producers about the correct practices and techniques for storage, processing, and conservation of oils to better improve the quality of the final product.

## 1. Introduction

Olives are the fruits of the olive tree (*Olea europaea* L.), one of the most popular and most consumed products in the Mediterranean region [1]. More than 96% of the Moroccan olive grove is made up of the Picholine variety [2]. It is characterized by its adaptability and the quality of its olives, which have a dual purpose: the production of olives and olive oil. According to the Olive Oil Quality Standard, the product must respect a set of physicochemical and organoleptic criteria that will help classify it into different categories [3].

Food quality depends on all its physicochemical, nutritional, and organoleptic properties. In particular, for olive oil, as for all vegetable products, quality originates from the choice of cultivars and the related cultural practices, as well as from the harvest time [4]. The choice of harvest techniques, postharvest operations, and oil extraction technologies is equally important [5]. After all, olive oil quality necessitates the observance and safeguard of the original product's intrinsic properties, even enhanced by a good extraction process [6].

Morocco has been established as one of the biggest olive oil producers in the world [7], due to its unique climate and soils. However, the cultivation, collection, and production processes have not seen any major improvements for the major part of this industry, resulting in huge differences in oil quality from one mill to the other. The optimal harvesting period is between the months of October and February, depending on the rainfall and the geographical location [8]. In the 2018/2019 season, olive cultivation benefited from an important rainfall, resulting in an early cultivation period toward the end of 2018. In traditional mills, the olives are harvested manually, sometimes over a relatively long period. In most cases, the extraction procedure does not differ much between mills, but the handling and storage of the oil are critical for its quality.

In order to evaluate the quality of the locally produced olive oils, this investigation was focused on studying the chemical composition of these oils from different traditional oil mills across Morocco. Physicochemical analyses such as free fatty acid (FFA), iodine value (IV), saponification value (SV), specific extinctions coefficients (E232, E270), chlorophyll content, carotenoid content, fatty acids (FAs), and total phytosterol composition were carried out. The outcome of this study will be extremely helpful to better understand the significance of traditional mills practices and will further help better the oil quality in these mills.

## 2. Materials and Methods

### 2.1. Sample

The study examined ten samples of Picholine olive oils (POOs) originating from traditional oil mills from the following cities: Beni Mella (BM), Taza (TZ), Zaouiat Cheikh (ZC), Errachidia (ERR), Missour (MI), Chichaoua (CH), Kelaât Sraghna (KS), Guercif (GR), Fquih Ben Saleh (FBS), and Shoul (SH), during the olive harvest season of 2018/2019. The geographical data of the cities are shown in Table 1.

After the harvest, the mature olives were processed within 24 hours according to the traditional system. Only fruits without any physical damage were extracted. The olives were cleaned of leaves and crushed, and the resulting olive paste was malaxed and pressed. The oil was decanted from the effluents of olive oil mills. After filtration, three samples (3 L bottle) were collected in each location and stored at 4°C in darkness using amber glass bottles until analysis.

### 2.2. Quality Parameters

FFA content, IV, SV, and extinction coefficients (E232 and E270) were determined according to AOCS recommended practices Ca 3a-63, Cd 1b-87, Cd 3b-76, and Ch 5-91, respectively [9]. FFA content was expressed as a percent of oleic acid, IV was expressed as mg I_2_/100g of oil, SV was expressed as mg KOH/1g of oil, and extinction coefficient (E232 and E270) was expressed as the specific extinction of a 1% (w/v) solution of oil in cyclohexane in 1 cm cell path length, using an LLG-uniSPEC 2 UV spectrometer.

### 2.3. Determination of Chlorophyll and Carotenoid Content

1 g of olive oil is dissolved in 100 mL of cyclohexane. After homogenization, absorbance is measured at 670 nm for chlorophylls and 470 nm for carotenoids [10]. The chlorophyll and carotenoid contents are calculated according to the following two formulas (equations (1) and (2)):

(1) $$\begin{array}{c} \text{Chlorophyll }\left(\text{mg}\cdot {\text{Kg}}^{-1}\right)=\frac{\left({\text{A}}_{670\;}\times \;10^{6}\right)}{\left(613\;\times 100\times \text{d}\right)}, \end{array}$$

(2) $$\begin{array}{c} \text{Carotenoid }\left(\text{mg}\cdot {\text{Kg}}^{-1}\right)=\frac{\left({\text{A}}_{470\;}\times \;10^{6}\right)}{\left(2000\;\times 100\times \text{d}\right)}. \end{array}$$

The chlorophyll content expressed in mg Alpha Pheophytin by kilogram of olive oil and the carotenoid content is calculated according to the following formula and expressed in mg Lutein by kilogram of olive oil.

### 2.4. Fatty Acids' Composition

The fatty acid methyl esters FAME composition was determined following the EEC/2568/91 protocol [11], by capillary gas chromatography (CGC), using a Varian CP-3800 (Varian Inc.) chromatograph equipped with an FID. A split injector was used, and the injected volume was 1 mL. The column used was a CP-Wax 52CB column (30 m × 0.25 mm i.d.; Varian Inc., Middelburg, The Netherlands). The conditions for the chromatographic operations were as follows: the carrier gas was helium and the total gas flow rate was 1 mL/min. The initial and final column temperature was 170°C and 230°C, respectively, and the temperature was increased by steps of 4°C/min. The injector and detector temperature was 230°C. Data were processed using a Varian Star Workstation v 6.30 (Varian Inc., Walnut Creek, CA, USA). Results were expressed as the relative percentage of each individual FA presents in the sample.

### 2.5. Phytosterols' Composition

The phytosterols were quantified according to the ISO 6799 [12] standard method using capillary gas chromatography (CGC) on an apolar column (Chroma pack) (30 m × 0.32 mm, DI : 0.25 *μ*m, phase: CPSIL8CB). The VARIAN CP-3800 chromatograph is equipped with a divider injector type 1079 (T : 300°C) and an FID (T : 300°C). The carrier gas is helium (flow: 1.5 mL/min).

### 2.6. Principal Component Analysis (PCA)

In this work, the main component analysis aims to establish the existence of a correlation between the different physicochemical parameters of the quality used of the olive oils (Picholine variety) on the one hand and between the chlorophyll content, carotenoid content, fatty acid, and phytosterol composition on the other hand. The main component analysis was realized on the results of physicochemical quality parameters (FFA, IV, SV, E232, and E270), the chlorophyll content, carotenoid content, polyunsaturated fatty acids (Linolenic (C18 : 3) and Linoleic (C18 : 2)), and total phytosterol composition, which represented the ten variables of POO originating from different regions. This method facilitates the interpretation of the fundamental factors contributing most to explain the variation in physicochemical quality parameters according to the geographic region of the samples and to investigate whether there was a correlation among the parameters' quality, chlorophyll content, carotenoid content, fatty acid, and phytosterol composition.

### 2.7. Correlation Matrix

The PCA was performed out on a matrix that resumes all the data of the different physicochemical parameters of the quality (FFA, IV, SV, E232, and E270), chlorophyll content, carotenoid content, fatty acids (Linolenic (C18 : 3) and Linoleic (C18 : 2)), and total phytosterol composition. The individuals are represented by the ten samples of Picholine olive oils originating from different regions.

### 2.8. Data Analysis

The Pearson correlation was used to study the correlation between all the physicochemical parameters' quality and all samples of Picholine olive oils of this study. The principal component analysis (PCA) was carried out to associate the variables with the samples from this study in a graphic representation by the XLSTAT 2014 software [13]. Data were expressed as mean ± standard error of the mean and were performed using IBM SPSS Statistics 21 software.

## 3. Results and Discussion

### 3.1. Quality Parameters

The FFA values of the studied samples are presented in Table 2. FFA values ranged between 0.59% and 2.74%. On the basis of these results, and according to the commercial standard of the IOC, the oils studied can be classified into three distinct categories [3]. Five of the studied samples (acidity lower than 0.8%) fall into the extra virgin olive oil class, namely, SH, ZC, GR, CH, CH, and BM. The second class is virgin olive oils with an FFA of <2%, and the samples TZ, MI, and FBS fall into this class. The third class is ordinary virgin olive oils with an FFA of 3.3% or less and samples KA and ERR are included in it. None of the studied samples was found to belong to the class of Lampante virgin olive oils, with an FFA of more than 3.3%.

Several factors, such as the maturity of the fruits, the harvesting method, and storage of the olives, can lead to lipases action and thus a high FFA content and acid value [14]. The results obtained in this study are lower than those reported by Benabid et al. [15], which were between 0.77% and 9.26% for olive oils from different olive-growing regions in Algeria. On the other hand, the values are high in comparison to those reported by Tanouti et al. [16], where FFA was found to be less than 0.8% for olive oils produced in eastern Morocco.

The results of the IV are shown in Table 2. The results are expressed in milligrams of iodine per 100 grams of oil (mg I_2_/100 g of olive oil).

It should be noted that the IV ranges from 86.51 mg *I*_2_/100 g olive oil for the TZ sample to 106.17 mg *I*_2_/100 g olive oil for the MI sample. However, SH, ERR, and CH samples reported IV values in the order of 89 mg *I*_2_/100 g olive oil. Finally, ZC, GR, FBS, and BM reported values in the order of 87 mg *I*_2_/100 g olive oil. Ragiab et al. [17] have reported an IV going from 71 to 97 mg *I*_2_/100 g of olive oil for Libyan olive oil in the western region.

The values of the saponification index are presented in Table 2. The results are expressed in milligrams of KOH per gram of oil (mg KOH/1 g olive oil). The SV of olive oils from the different samples studied ranges from 197.89 mg KOH/1 g olive oil for the MI sample to 224.26 mg KOH/1 g olive oil for the TZ sample. However, BM, FBS, and ERR have SV values that are close to 204 mg KOH/1 g olive oil while CH and ZC samples have values in the order of 210 mg KOH/1 g of olive oil. These values of SV are similar to those reported by Ragiab et al. [17].

The UV absorption at 232 nm (E232) and 270 nm (E270) is useful rapid methods to evaluate the presence of primary and secondary oxidation products, respectively.  Table 3 shows that the values of the specific extinctions E232 and E270 are within the limits set by the IOC for virgin olive oils [3], which must be between 0.22 and 2.50. The ZC sample has the highest value of E232 in the order of 2.25; this value remains below the limit set by the IOC (<2.5). Many factors may explain these results, such as late harvesting of olives, excessive exposure of olives and extracted oil to oxygen and sunlight, and even heating of the pulp during crushing [18]. The specific extinction at 232 and 270 nm of oil reflects its oxidation state. The higher the 232 nm extinction value is, the more peroxidized it is. Similarly, the higher the 270 nm extinction value is, the richer in secondary oxidation products, and the poorest its conservation properties are [10].

### 3.2. Determination of Chlorophyll and Carotenoid Content

**Figure 1:**
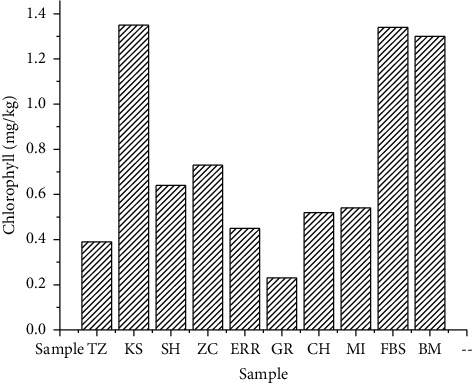
Chlorophyll content of the studied samples.

**Figure 2:**
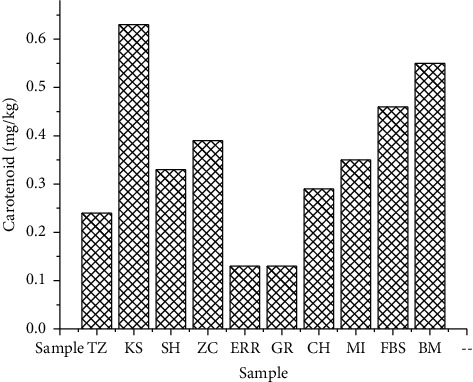
Carotenoid content of the studied samples.

The concentrations obtained for chlorophyll and carotenoid in the samples studied, expressed in mg/kg, are presented in Figures 1 and 2.

The chlorophyll contents, for most of the studied samples, are less than 2 mg/kg (Figure 1). Lower chlorophyll values are desired in vegetal oils to avoid prooxidation and ensure proper conservation of the oils [19]; this further accentuates the importance of producing olive oils removing leaves during the oil extraction process. Indeed, at the start of olive maturity, the concentration of chlorophylls is elevated. This value decreases continuously with the maturity of the olives. The yellow oil color is due to the degradation of chlorophylls into pheophytins [20].

As shown in Figure 2, the carotenoid content varied with concentrations between 0.13 and 0.63 mg/kg. In fact, the GR sample represents the lowest concentration of total chlorophyll and carotenoids (0.23 and 0.13 mg/kg, respectively). The KS sample contained the highest concentration of chlorophyll and carotenoids (1.35 and 0.63 mg/kg, respectively). The pigment contents in our samples are lower than those reported by Minguez-Mosquera et al. [21], with quantities of 1-54.4 mg/kg for chlorophylls and 2.6-22.5 mg/kg for carotenoids. In another study, Šarolić et al. reported chlorophyll and carotenoid content of Croatian olive oils ranging from 3.86 to 4.75 mg/kg and from 1.89 to 2.06 mg/kg, respectively [22]. However, Guerfel et al. [23] and Issaoui et al. [24] reported similar levels and explained that the level of pigments in the oil depends on many factors, such as the olive cultivar (Picholine in our case) and the cultivation location.

### 3.3. Fatty Acids' Composition

The results obtained from the GC analysis are presented in Table 3. The results obtained for the 10 studied samples show that the fatty acid composition of the olive oils analyzed conforms to the standards fixed by the IOC [25]. The percentages of oleic acid (C18 : 1) vary between 56.95% for the MI sample and 80.38% for the TZ sample while the percentages of linoleic acid (C18 : 2) vary between 6.86% for the TZ sample and 25.45% for the MI sample; these two fatty acids are the major ones. Second to those two acids comes palmitic (C16 : 0) and stearic (C18 : 0) acids, with the highest values obtained for the ERR sample with 10.84%, and KA and GR samples with 2.98%, respectively. The minor fatty acids with percentages not exceeding 4% are palmitoleic, stearic, linoleic, arachidic, and gadoleic acids. Margaric acid and margaroleic acid were detected as traces with percentages below 0.2%.

The presence of PUFAs: linoleic acid (C18 : 2) with a high percentage compared to other unsaturated fatty acids can be explained by the presence of an enzyme, oleate desaturase, which transforms oleic acid (C18 : 1) into linoleic acid (C18 : 2) during fruit maturation [26, 27]. The percentages of oleic acid in the olive oils studied are similar to the values reported by Abu-Reidah et al. [28] and Harhar et al. [27], which are ranging from 67.24 to 72.27% for Palestinian oils. However, they are slightly higher than the values reported by Issaoui et al. [24] for Tunisian oils (54.6 to 66.8%). It should also be mentioned that the fatty acid composition obtained reveals a predominance of monounsaturated fatty acids. The percentage of unsaturated fatty acids (UFAs) varies slightly and depends on the samples studied. It varies between 85.94 for ERR and 88.94% for GR. Similarly, the percentage of saturated fatty acids (SFAs) varies from 11.05 for TZ to 14.02% for ERR. The ratio of UFAs/SFAs also shows a variation with the samples studied. This ratio ranges from 6.13 for ERR to 8.05 for TZ. The higher the ratio, the more the stability to autooxidation and the high nutritional quality the oil has [29, 30].

### 3.4. Phytosterols' Composition

**Figure 3:**
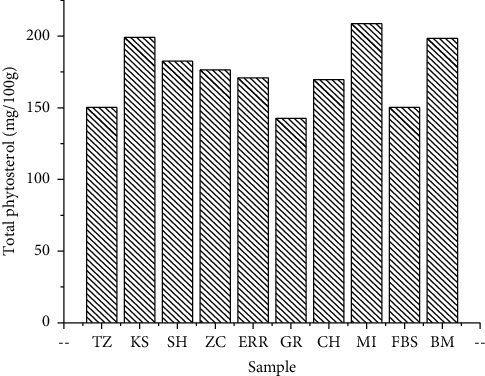
Total phytosterol content of the studied samples.

Phytosterols' composition is an important indicator for oil quality and authenticity [31]. The results of the total phytosterol content assay are shown in Figure 3, and the composition of phytosterols is shown in Table 4. The results are expressed in milligrams by 100 grams of oil (mg/100 g).

The results obtained in total phytosterols from the 10 studied samples show that olive oils are rich in total phytosterols with variability between 142.68 and 208.72 mg per 100 g of oil (Figure 3). The highest value was recorded for the IM sample (208.72 mg/100 g), while the lowest value was obtained for the GR sample (142.68 mg/100 g). Two different types of phytosterols represent about 90% of the total phytosterol content, namely, *β*-sitosterol and Δ-5-avenasterol.

The results obtained from the gas chromatography assay are presented in Table 4. The results obtained for the 10 studied samples show that the phytosterol composition meets the required IOC standards [25], with the exception of the TZ sample which has a high cholesterol value of 7.60%.

The phytosterols' composition varies from one city to another. Indeed, the percentage of *β*-sitosterol varies between 69.90% for the MI sample and 86.40% for the BM sample while the percentages of Campesterol vary between 2.65% for the TZ sample and 10.48% for the MI sample. The high *β*-sitosterol content in olive oil is most likely responsible for the great preservation ability it has [31, 32].

The percentages of Δ-5-avenasterol range from 3.74% for the MI sample to 9.52% for the ERR sample. Interestingly, Δ-5-avenasterol is known to act as an antioxidant and antipolymerization agent in frying oils [33, 34].

Additionally, stigmasterol and Δ-7-stigmasterol were identified with values ranging from 0.71% for the CH sample to 9.84% for the MI sample and from 0.13% for the GR sample to 2.25% for the MI sample, respectively.

### 3.5. Correlation Matrix

Table 5 presents the Pearson correlation that helps analyze the relationships between the different variables tested in this study.  Table 6 presents the *p* values of the correlation matrix coefficient between all variables. We can see that there is a significant positive correlation (*p* value <0.05) between IV and E270 (*r*^2^ = 0.668). Another positive correlation (*p* value <0.05) between IV and total phytosterol (*r*^2^ = 0.690) can be observed. There was also a moderately positive correlation (*p* value <0.05) between E270 and PUFAs (Linolenic (C18 : 3) (*r*^2^ = 0.682) and Linoleic (C18 : 2) (*r*^2^ = 0.670)). Moreover, another significant positive correlation (*p* value <0.05) has been observed between total phytosterol and Linolenic C18 : 3) (*r*^2^ = 0.741) and Linoleic (C18 : 2) (*r*^2^ = 0.713), respectively.

A high positive correlation (*p* value <0.0001) was observed between the PUFAs (*r*^2^ = 0.987). Also, a highly significant positive correlation (*p* value <0.0001) between IV and PUFAs (Linolenic (C18 : 3) (*r*^2^ = 0.990) and Linoleic (C18 : 2) (*r*^2^ = 0.994)) was observed. Our results are consistent with those obtained by Samuel et al. [35], where it was indicated that the high iodine value of fluted pumpkin seed oil indicates that the oil is rich in polyunsaturated fatty acids, which enhances the nutritional value of food products. Aremu et al. [36] showed that the lower the iodine value, the lesser the number of unsaturated bonds.

### 3.6. Principal Component Analysis (PCA)

**Figure 4:**
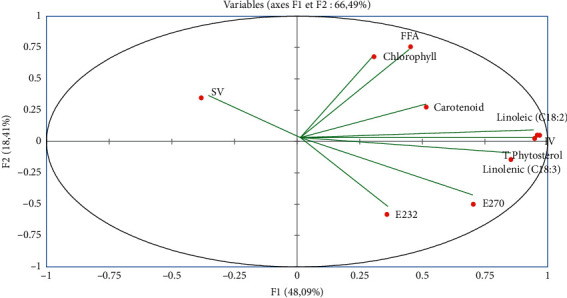
PCA factorial plan carried out on the values (FFA, IV, SV, E232, E270, chlorophyll, carotenoid, total phytosterols, and PUFAs (Linolenic (C18 : 3) and Linoleic (C18 : 2)) of the different samples of POOs.

The different physicochemical quality parameters and the results of the chlorophyll content, carotenoid content, polyunsaturated fatty acids' (Linolenic (C18 : 3) and Linoleic (C18 : 2)) composition, and total phytosterol composition are considered as variables. They are projected by the PCA on the F1-F2 factorial plan (Figure 4). The first main component (F1) shows 48.6% of the total information, and the second main component (F2) explains 18.41%. The cumulative percentage of the two first principal components is 66.49%; consequently, its linear combination is representative of the variables because it is greater than 50%. Hence, the first two axes are suitable to represent the information as a whole.

Figure 4 shows the plane formed by axes F1 and F2, giving the correlation between the variables. The F1 axis is mainly constructed by the positive correlation between the PUFAs (Linolenic (C18 : 3), Linoleic (C18 : 2)), total phytosterol composition, E232, E270, and carotenoid content; also it is formed by SV. Axis F2 is formed by the positive correlation between the FFA and the chlorophyll content.

**Figure 5:**
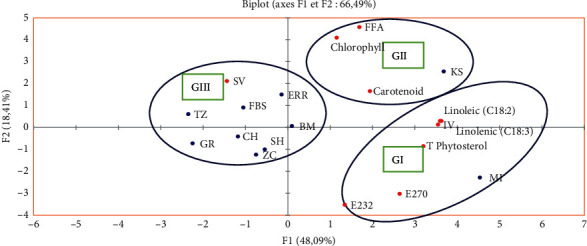
Projection of individuals on the factorial plan (F1 × F2). GI: Group I; GII: Group II; GII: Group III.

Figure 5 shows that 10 individuals are spread (of POO) into 3 groups.

Group I is made up of one individual sample Missour (MI). It is characterized by the high content of the total phytosterol, carotenoid, and the strong value of the iodine value (IV), which is characterized by the higher values of PUFAs (Linolenic (18 : 3) and Linoleic (C18 : 2)). Moreover, they have a high value of extinction coefficient (E232 and E270).

Group II is formed by the sample KS native from the Kelaât Sraghna (KS). It is characterized by the high content of chlorophyll, carotenoid content, and the strong value of FFA. Also, they have a medium value of PUFAs and IV.

Group III consists of 8 samples of POO originating from traditional oil mills from the following cities: Fquih Ben Saleh (FBS), Shoul (SH), Taza (TZ), Zaouiat Cheikh (ZC), Chichaoua (CH), Guercif (GR), Beni Mella (BM), and Errachidia (ERR). The saponification value (SV) of the nine samples is significantly higher than that of the other samples, but the levels of PUFAs (Linolenic (C18 : 3) and Linoleic(C18 : 2)) are lower than those of the other samples of POO in groups I and II, which shows the low value of IV.

## 4. Conclusion

The study of the quality parameters of Picholine olive oils from different traditional olive mills for different cities of Morocco allowed us to classify these oils into different classes. However, none of the studied samples can be qualified as an extra virgin olive oil. The high acidity values obtained are mainly due to the failure to comply with good harvesting, crushing, and storage practices for olive oils. The fatty acids' composition revealed that the olive oils studied are rich in unsaturated fatty acids: C18 : 1, C18 : 2, and C18 : 3. Indeed, the ratio of unsaturated fatty acids and saturated fatty acids is high and ranges from 6.42 to 6.76. As a highly consumed product, the conservation conditions of olive oils have a direct impact on human health, thus we need to improve the local production and grant it a higher priority. The best and most direct way for this purpose is to inform and spread awareness of the correct practices for storage, processing, and conservation of olive oils.

## Figures and Tables

**Table 1:** Geographical data of Picholine olive oil collection sites.

Location	Altitude (m)	Average temperature (°C)	Rainfall (mm)
BM	500	18.3	493
TZ	550	17.9	563
ZC	765	17.7	666
ERR	1039	19.2	127
MI	908	16.3	190
CH	339	18.6	242
KS	400	19.1	278
GR	378	18.5	222
FBS	432	18.9	483

**Table 2:** Quality parameters of the studied olive oils.

Sample	FFA (%)	IV (mg I_2_/100 g olive oil)	SV (mg KOH/1 g olive oil)	E232	E270
TZ	1.39 ± 0.02	86.51 ± 0.04	224.26 ± 0.08	0.38 ± 0.02	0.16 ± 0.01
KS	2.74 ± 0.02	105.39 ± 0.08	208.13 ± 0.06	0.61 ± 0.03	0.14 ± 0.01
SH	0.59 ± 0.01	89.48 ± 0.04	200.98 ± 0.04	1.17 ± 0.04	0.13 ± 0.01
ZC	0.64 ± 0.01	87.73 ± 0.04	210.23 ± 0.06	2.25 ± 0.04	0.13 ± 0.01
ERR	2.63 ± 0.03	89.56 ± 0.06	204.62 ± 0.04	0.86 ± 0.02	0.16 ± 0.01
GR	0.78 ± 0.01	87.98 ± 0.04	199.01 ± 0.04	0.23 ± 0.01	0.11 ± 0.01
CH	0.76 ± 0.01	89.69 ± 0.06	210.93 ± 0.06	0.85 ± 0.02	0.13 ± 0.01
MI	1.25 ± 0.02	106.17 ± 0.07	197.89 ± 0.04	1.76 ± 0.03	0.15 ± 0.02
FBS	1.13 ± 0.01	87.88 ± 0.04	204.48 ± 0.06	0.79 ± 0.02	0.13 ± 0.01
BM	0.70 ± 0.01	87.19 ± 0.06	204.06 ± 0.06	1.14 ± 0.04	0.16 ± 0.01
Standard (IOC)	<0.8			<2.5	<0.22

All values are the mean of three replicates ± standard deviation of the mean.

**Table 3:** Fatty acid composition of the studied samples.

FAs (%)	TZ	KS	SH	ZC	ERR	GR	CH	MI	FBS	BM	Standard (IOC)
Palmitic (C16 : 0)	8.06	10.08	9.41	8.18	10.84	7.75	9.10	10.40	8.55	10.27	7.5–20.0
Palmitoleic (C16 : 1)	0.55	0.43	0.72	0.62	0.95	0.56	0.64	0.50	0.62	0.75	0.3–3.5
Stearic (C18 : 0)	2.62	2.98	2.43	2.72	2.86	2.98	2.19	2.92	2.57	2.20	0.5–5.0
Oleic (C18 : 1)	80.38	58.22	75.22	78.86	71.42	78.86	76.08	56.95	78.32	76.53	55.0–83.0
Linoleic (C18 : 2)	6.86	24.57	10.61	8.06	12.16	8.36	10.46	25.45	8.49	8.51	2.5–21.0
Linolenic (C18 : 3)	0.70	2.94	0.90	0.81	1.04	0.73	0.83	3.02	0.76	1.01	0.0–1.0
Arachidic (C20 : 0)	0.33	0.34	0.26	0.28	0.28	0.29	0.24	0.33	0.27	0.29	<0.60
Gadoleic (C20 : 1)	0.36	0.30	0.35	0.36	0.31	0.37	0.38	0.28	0.35	0.33	<0.50
SFAs	11.05	13.45	12.13	11.22	14.02	11.06	11.56	13.71	11.42	12.80	
UFAs	88.93	86.51	87.85	88.78	85.94	88.94	88.45	86.16	88.59	87.20	
UFAs/SFAs	8.05	6.43	7.24	7.91	6.13	8.04	7.65	6.28	7.76	6.81	

**Table 4:** Phytosterol composition of the studied samples.

Phytosterols (%)	TZ	KS	SH	ZC	ERR	GR	CH	MI	FBS	BM	Standard (IOC)
Cholesterol	7.60	0.05	0.06	0.07	0.03	0.06	0.05	0.20	0.07	0.08	<0.5
Campesterol	2.65	3.04	3.10	3.01	3.09	3.23	3.05	10.48	3.19	3.00	<4.0
Stigmasterol	1.22	2.71	0.72	1.09	1.20	1.01	0.71	9.84	0.87	1.01	< Campesterol
*β*-sitosterol	77.63	82.75	86.23	85.92	80.84	83.86	85.17	69.90	84.44	86.40	
Δ-5-Avenasterol	8.65	9.08	7.57	7.40	9.52	9.38	8.6	3.74	9.07	7.07	
Δ-7-stigmasterol	0.18	0.14	0.18	0.21	2.23	0.13	0.28	2.25	0.20	0.23	<0.5
Δ-7-Avenasterol	0.31	0.30	0.29	0.31	0.27	0.34	0.30	1.02	0.27	0.35	
Others	1.75	1.92	1.84	2.00	2.02	1.99	1.84	2.58	3.86	1.85	

**Table 5:** Pearson's correlation matrix coefficient between the variables: quality parameters (FFA, IV, SV, E232, and E270), chlorophyll, carotenoid, total phytosterols, Linolenic (C18 : 3), and Linoleic (C18 : 2) acids of the different samples of POOs.

Variables	FFA	IV	SV	E232	E270	Chlorophyll	Carotenoid	T. phytosterol	Linolenic (C18 : 3)	Linoleic (C18 : 2)
FFA	1									
IV	0.490	1								
SV	0.121	−0.302	1							
E232	−0.306	0.173	−0.213	1						
E270	0.058	0.668	−0.327	0.426	1					
Chlorophyll	0.564	0.112	−0.126	0.028	−0.165	1				
Carotenoid	0.082	0.395	−0.013	0.241	0.019	0.523	1			
T. phytosterol	0.177	0.690	−0.324	0.553	0.561	0.308	0.585	1		
Linolenic(C18 : 3)	0.505	0.990	−0.288	0.195	0.682	0.176	0.451	0.741	1	
Linoleic(C18 : 2)	0.535	0.994	−0.335	0.174	0.670	0.170	0.367	0.713	0.987	1

The values in bold are different from 0 at a significance level alpha = 0.05.

**Table 6:** *p* values of the correlation matrix coefficient between all variables.

Variables	FFA	IV	SV	E232	E270	Chlorophyll	Carotenoid	T. phytosterol	Linolenic (C18 : 3)	Linoleic (C18 : 2)
FFA	0									
IV	0.151	0								
SV	0.740	0.396	0							
E232	0.389	0.632	0.554	0						
E270	0.873	0.035	0.356	0.219	0					
Chlorophyll	0.089	0.758	0.728	0.938	0.648	0				
Carotenoid	0.823	0.259	0.971	0.503	0.958	0.121	0			
T. phytosterol	0.625	0.027	0.362	0.098	0.091	0.386	0.076	0		
Linolenic(C18 : 3)	0.136	<0.0001	0.420	0.589	0.030	0.626	0.191	0.014	0	
Linoleic(C18 : 2)	0.111	<0.0001	0.344	0.631	0.034	0.639	0.297	0.021	<0.0001	0

The values in bold are different from 0 at a significance level alpha = 0.05.

## Data Availability

The data used to support the findings of this study are available from the corresponding author upon request.
